# Tuberculosis in Jugo-Slavia

**Published:** 1920-10-23

**Authors:** 


### TUBERCULOSIS IN JUGO-SLAVIA.

By A CORRESPONDENT.

In the course of a six months' tour on the Continent I have visited France, Austria, Germany, Hungary, Czecho-Slovakia, Rumania, and Jugo-Slavia, and in the matter of physique I have little hesitation in giving the palm to the last-named nation. Indeed, the Jugo-Slavs impressed me as the finest-built men I had ever seen, not even excepting the Anzacs. Yet, in spite of such fine physique, the population is riddled with tuberculosis. How has this come about? The prevalence of the disease cannot, I think, be attributed mainly to underfeeding or to the hardships of war. Famine afflicted the country for only a comparatively short time, and at present Jugo-Slavia is one of the richest agricultural countries in the world, and one of the best fed; while other nations equally exposed to the hardships of war are not equally affected by tuberculosis. Nor do I think the prevalence of the disease can be attributed to special racial susceptibility. It is due mainly, I think, to overcrowding, to dirt, and to deficient hygiene. Jugo-Slavia has reached that stage of civilisation where urbanisation, with its overcrowding, its sedentary occupations, and its social intercourse, gives opportunity for infection; and it has not yet reached that stage where the dangers of infection are duly appreciated and precautions duly practised. The ravages which tuberculosis is making in Serbia can surprise no one who has travelled in that country.

To give an instance of what I mean: I travelled by steamer down the Danube from Baja, on the Hungarian frontier, to Belgrade. The little steamer was crammed with all sorts and conditions of passengers, some of them professional men and some of them peasants. At meal-times the little low-roofed, dirty dining-saloon was packed with diners, and at night the narrow, dirty corridors were heaped with huddled forms. In the saloon the windows were kept severely closed, and before meals were finished the atmosphere was more malodorous and foul than any atmosphere I have ever experienced elsewhere. In the corridors at night the smell of human bodies was sickening. That in itself would be unhygienic enough; but there were aggravating factors. There were only two lavatories, both out of order, and the stench pervaded the whole boat. Worse still, the habit of expectoration is indulged in by the Balkan peoples to an extraordinary extent. Most of the passengers seemed to be suffering from nasal, laryngeal catarrh, and the spitting was incessant. At dinner my neighbour, who was evidently consumptive, expectorated copiously between his feet and mine. In the morning I sat and noticed how every man who came up the gangway signalised his arrival by spitting on the deck.

The boat, as I have mentioned, was overcrowded, and at the end of forty-eight hours I doubt whether there was a square inch of deck or corridor-floor which was not contaminated. Not only so, but the canvas covering baskets of fruit and of fish was also befouled. The conditions were so disgusting that I found a comparatively clean place near the funnel, and stayed there most of the day. At least 5 per cent. of the passengers were consumptive, and I can conceive of no conditions more favourable for infection. The decks and the corridors, where they lay huddled up in rugs, were simply covered with microbes. It would, of course, have been perfectly easy to spit into the Danube, but the people had neither æsthetic feelings nor hygienic knowledge, and spat just where it was easiest to spit.

A boat in such a condition is a danger to every passenger travelling on it, and every trip north and south it must carry death with it. Railway trains, railway stations, public conveyances, hotels, and restaurants are in the same way sources of infection. People lie asleep along dirty train corridors or on dirty station floors; they are subjected to exhausting conditions; they breathe foul air; they are exposed to infection; and the wonder is that the whole nation is not tubercular.

It is time that the Balkan nations learned hygiene, for under the present conditions of overcrowding the habit of promiscuous expectoration is likely enough to more than decimate them.

